# Editorial: Enhancing plant health through silicon supplementation under nutritional stress

**DOI:** 10.3389/fpls.2026.1834639

**Published:** 2026-04-17

**Authors:** Kamilla Silva Oliveira, Dilier Olivera-Viciedo, Renato de Mello Prado, Jonas Pereira de Souza Júnior

**Affiliations:** 1 Department of Soil Science, Faculty of Agricultural and Veterinary Sciences, São Paulo State University (UNESP), Jaboticabal, Brazil; 2 Instituto de Ciencias Agroalimentarias, Animales y Ambientales, Universidad de O’Higgins, Rancagua, Chile; 3 Citrus Research and Education Center, University of Florida, Lake Alfred, FL, United States

**Keywords:** abiotic stress, beneficial element, mechanisms, mitigation, nutritional disorders

## 1 Silicon as a tool to mitigate nutritional stress in plants

Silicon (Si) is increasingly recognized as a beneficial element that improves plant resilience against a broad spectrum of biotic and abiotic stresses (Epstein, 1999; Tombeur et al., 2023). Although it has not historically been classified as essential, recent reconsiderations of the plant nutrient concept have proposed the inclusion of beneficial elements such as Si, considering their consistent and significant contributions to plant performance and stress tolerance (Brown et al., 2022).

Nutritional stress, arising from nutrient deficiency or toxicity, represents a major constraint on plant growth and productivity. Beyond soil nutrient availability, its severity may be amplified by unfavorable environmental conditions, thereby compounding the detrimental effects of other stresses on plant development (Dutta et al., 2024; Wang et al., 2025). Silicon has been shown to alleviate nutritional stress–induced damage in plants and to modulate macro- and micronutrient concentrations (Costa et al., 2024; Khan et al., 2023; Prado, 2023; Souza Junior et al., 2025). Nevertheless, the mechanisms underlying these effects remain insufficiently understood, particularly at the molecular level and in relation to their interaction with other stresses.

**Figure 1:**
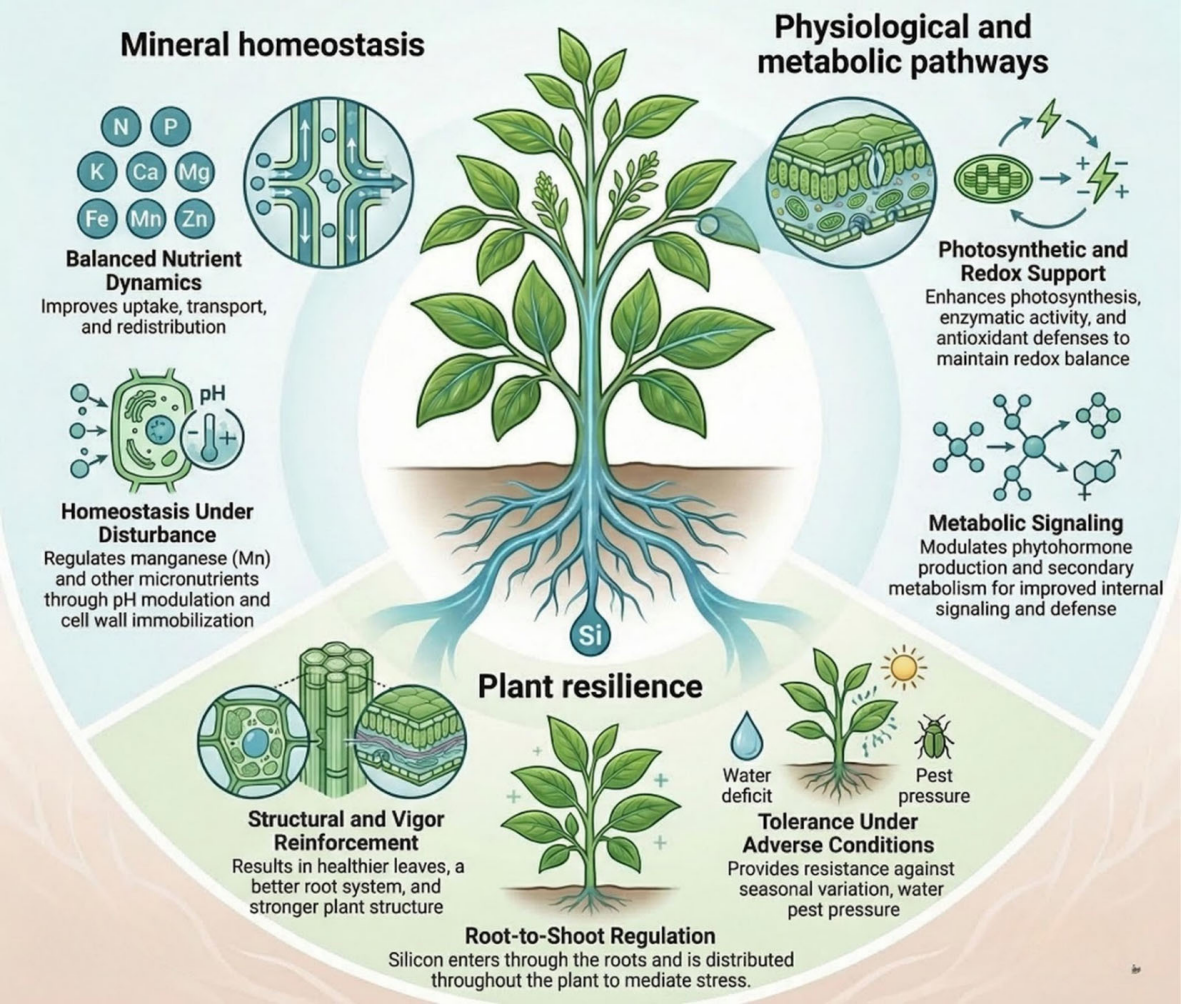
Conceptual overview of the role of silicon (Si) in enhancing plant resilience under nutritional stress. Silicon contributes to mineral homeostasis by regulating nutrient uptake, transport, and balance under deficiency or toxicity conditions. It also modulates physiological and metabolic processes, including photosynthesis, redox balance, and signaling pathways. Additionally, Si improves nutrient-use efficiency and supports the integration of plant responses to adverse conditions. Together, these mechanisms highlight the multifaceted role of Si in mitigating nutritional disorders and promoting plant resilience.

In this context, the present Research Topic compiles contributions that deepen our understanding of the role of Si in stress alleviation and the regulation of plant nutritional dynamics. The studies collectively underscore the multifaceted nature of Si-mediated responses under conditions of nutritional disturbance, highlighting its contribution to mineral homeostasis, the modulation of physiological and metabolic pathways, and the reinforcement of plant resilience under adverse conditions (**Figure 1**).

## 2 Silicon regulation of nutrient homeostasis and mineral balance

In this Research Topic, Coquerel et al. showed that Si influences key physiological and molecular processes involved in nodulation and nitrogen (N) acquisition, while also stimulating the uptake of additional nutrients. Notably, they provided the first evidence that Si differentially modulates the proteome of root host cells colonized by symbiotic bacteria, ultimately promoting greater shoot biomass in *Trifolium incarnatum* L. Collectively, these results deepen our understanding of the interplay between Si and N nutrition in plants.

Another key aspect relates to the role of Si in preserving nutritional homeostasis across different nutrient availability scenarios. Previous studies have already underscored its contribution to nutritional balance under limiting conditions (Araújo et al., 2022; Costa et al., 2025). In the review by Hailai et al., the authors examine the mechanisms by which Si regulates manganese (Mn) homeostasis, particularly under conditions of toxicity. Proposed mechanisms include modulation of rhizosphere pH, stimulation of Mn-reducing microorganisms, alterations in root exudation, potential regulation of Mn transporters, and immobilization of Mn in the leaf apoplast through interactions with cell wall components such as lignin and callose, a mechanism previously proposed for other micronutrients (Pavlovic et al., 2021). In contrast, under Mn deficiency, these mechanisms remain largely unresolved despite evidence of enhanced Mn uptake and use efficiency, highlighting the need for deeper molecular investigation. Taken together, the mitigation of oxidative stress appears to be a recurring mechanism underlying Si action under distinct conditions of Mn imbalance.

## 3 Silicon-mediated nutritional resilience

Nutritional disorders can act both as drivers and consequences of environmental and biotic stresses, while also representing a central factor in the modulation of plant resilience (Khan et al., 2023; Prado et al., 2024). Within this framework, the role of Si in regulating mineral nutrition under stress emerges as a promising basis for the development of innovative and sustainable strategies in plant nutrition. Fajardo et al. showed that Si application alleviates the negative effects of seasonal variation in citrus cultivation, a condition that typically restricts nutrient uptake due to environmental and physiological limitations. Silicon enhanced nutrient acquisition during critical cultivation periods and promoted plant growth, particularly under low P availability. A comparable response appears to occur under pest and disease pressure, as reported by Verma et al., where Si increased nutrient acquisition and improved nutritional efficiency during biotic stress in coordination with signaling pathways.

These beneficial interactions between Si and mineral nutrition, even under stress conditions, have also fueled interest in the development of more efficient fertilization strategies. In this regard, Alharbi et al. demonstrated that the application of Si- and K-based nanoparticles alleviated the effects of water deficit in sorghum, enhancing plant growth and increasing the accumulation of K and other nutrients, particularly P. Similarly, El-Mahrouk et al. reported that the combined application of Si and Zn nanoparticles with pollen extract reduced the NPK requirement of *Salvia officinalis*. Taken together, these findings highlight the potential of Si-based technologies, including nanofertilizers, as promising tools for enhancing nutrient-use efficiency and expanding the use of Si in agricultural systems.

## 4 Physiological and metabolic mechanisms of silicon

Beyond the direct role of Si in nutrient regulation under nutritional disorders, the beneficial element may enhance plant physiological performance by promoting nutrient uptake and translocation, thereby supporting biosynthetic activity, enzymatic activation, and electron transport processes that are fundamental to photosynthesis, as highlighted by Mukarram et al. These effects may further extend to secondary metabolism, including the production of phytohormones involved in defense against herbivory stress in rice, as investigated by Osibe et al. Such responses may, in turn, be facilitated by improved nutritional balance, since plant nutrition provides the metabolic basis for the biosynthesis of secondary metabolites (Rubio et al., 2009).

## 5 Research future directions

Despite the important advances highlighted in this Research Topic, several key questions remain open regarding the role of Si in improving plant performance under nutritional stress. Future research should move beyond the description of beneficial effects and seek to integrate molecular, physiological, biochemical, and agronomic approaches in order to better explain how Si regulates nutrient homeostasis under conditions of deficiency, toxicity, and combined stresses. Particular attention should be given to the coordination between Si and nutrient transport, signaling pathways, redox metabolism, phytohormonal regulation, and plant–microbe interactions, as these processes appear to act together in shaping plant resilience.

In addition, more studies are needed to determine how Si responses vary across species, cultivars, environmental conditions, and sources or forms of Si supply, including conventional fertilizers and emerging nanotechnologies. Expanding this knowledge will be essential for clarifying the mechanisms of action of Si and for translating these advances into practical strategies capable of improving nutrient-use efficiency, crop productivity, and sustainability in agricultural systems increasingly exposed to nutritional and environmental challenges.
